# Breed of origin of alleles and genomic predictions for crossbred dairy cows

**DOI:** 10.1186/s12711-021-00678-3

**Published:** 2021-11-06

**Authors:** Jón H. Eiríksson, Emre Karaman, Guosheng Su, Ole F. Christensen

**Affiliations:** Center for Quantitative Genetics and Genomics, Aarhus University, 8830 Tjele, Denmark

## Abstract

**Background:**

In dairy cattle, genomic selection has been implemented successfully for purebred populations, but, to date, genomic estimated breeding values (GEBV) for crossbred cows are rarely available, although they are valuable for rotational crossbreeding schemes that are promoted as efficient strategies. An attractive approach to provide GEBV for crossbreds is to use estimated marker effects from the genetic evaluation of purebreds. The effects of each marker allele in crossbreds can depend on the breed of origin of the allele (BOA), thus applying marker effects based on BOA could result in more accurate GEBV than applying only proportional contribution of the purebreds. Application of BOA models in rotational crossbreeding requires methods for detecting BOA, but the existing methods have not been developed for rotational crossbreeding. Therefore, the aims of this study were to develop and test methods for detecting BOA in a rotational crossbreeding system, and to investigate methods for calculating GEBV for crossbred cows using estimated marker effects from purebreds.

**Results:**

For detecting BOA in crossbred cows from rotational crossbreeding for which pedigree is recorded, we developed the AllOr method based on the comparison of haplotypes in overlapping windows. To calculate the GEBV of crossbred cows, two models were compared: a BOA model where marker effects estimated from purebreds are combined based on the detected BOA; and a breed proportion model where marker effects are combined based on estimated breed proportions. The methods were tested on simulated data that mimic the first four generations of rotational crossbreeding between Holstein, Jersey and Red Dairy Cattle. The AllOr method detected BOA correctly for 99.6% of the marker alleles across the four crossbred generations. The reliability of GEBV was higher with the BOA model than with the breed proportion model for the four generations of crossbreeding, with the largest difference observed in the first generation.

**Conclusions:**

In rotational crossbreeding for which pedigree is recorded, BOA can be accurately detected using the AllOr method. Combining marker effects estimated from purebreds to predict the breeding value of crossbreds based on BOA is a promising approach to provide GEBV for crossbred dairy cows.

## Background

Crossbreeding is common practice in many livestock production systems, where production animals have parents from different breeds, lines or populations. Three-breed rotational crossbreeding systems have been recommended for dairy cattle [1, 2]. In rotational crossbreeding, crossbred cows with different breed combinations are present within the same herds and the crossbred cows are potential dams for the next generation. Thus, knowing the breeding values of crossbred cows is useful for selection. This contrasts with the situation in terminal crossbreeding, which is common in chicken and pig production, where production groups are typically uniform and the breeding value of crossbred animals is not of interest per se. Thus, providing genomic estimated breeding values (GEBV) of crossbred cows will be valuable for within-herd selection and would give producers the possibility of using the benefits of both crossbreeding and GEBV of cows simultaneously.

The genomic evaluation of crossbred animals and across populations may lead to challenges that are not experienced when it is performed within uniform populations [3]. Genomic evaluations are based on the effects of genetic markers, which deviate between breeds and populations, partly because of differences in linkage disequilibrium between the genetic markers and the quantitative trait loci (QTL), which are generally smaller across breeds than within breeds [3, 4]. Thus, in the case of crossbred animals, the same marker allele may have a different effect according to the breed of origin of the allele (BOA) [4, 5].

For the pedigree-based genetic evaluation of crossbred animals, García-Cortés and Toro [6] presented a model where the breeding values of crossbred animals were divided into breed-specific and breed segregation terms. Strandén and Mäntysaari [7] proposed a random regression approximation of that model so that it could include genomic information. Makgahlela et al. [8] tested the model, without accounting for the breed segregation terms, for genomic prediction of the admixed Red Dairy Cattle (RDC) population. Their results indicated a slight increase in prediction accuracy compared to the use of a genomic model that did not take breed structure into account [8]. VanRaden et al. [9] demonstrated that genomic predicted transmitting abilities of crossbred dairy cows could be calculated based on the same concept of partitioning the breeding value of crossbreds into breed-specific purebred terms. However, instead of using breed proportions based on the registered pedigree as done by Makgahlela et al. [8], VanRaden et al. [9] estimated base breed representation (BBR) from genotypes of the crossbred and used it as a measure of the proportion of genome originating from each breed. These proportions were used as weights for combining genomic predicted transmitting abilities based on results from within-breed genomic evaluations. The large reference populations that have been established for purebred genomic evaluations provide accurate estimates of marker effects, and thus they could be used to calculate GEBV of crossbred cows. The method presented in VanRaden et al. [9] is based on the approximation that markers at each locus come from the pure breeds in equal proportions. This only holds for the first generation of crossbreds (F1), because for more complex crosses, the local ancestry varies throughout the genome, and at each locus the two alleles are from only one or two breeds. In addition, these proportions do not consider from which pure breeds the two alleles originate, which is relevant for all heterozygous loci. Thus, models that account for the BOA specific effects at each locus could be more appropriate.

Models with BOA specific effects, hereafter denoted as “BOA models”, have been tested on both simulated and empirical data from terminal crossbreeding production systems [4, 10, 11]. These models are able to account for the difference in effects of markers due to differences in linkage disequilibrium between markers and QTL. In a simulation study, Ibánẽz-Escriche et al. [4] found that BOA models could outperform the models using common marker effects across breeds only if the breeds included were distantly related, the number of markers was small, and the training set was large. Sevillano et al. [10] studied three-way crosses in pigs and concluded that BOA models are only justified in cases where the correlation between crossbred and purebred performance is low, the heritability of the trait is low, and the breeds involved are distantly related. However for a trait with a low heritability, Xiang et al. [11] tested a model with partial genomic relationship matrices that accounted for BOA for the evaluation of crossbred performance in two-way crossbred pigs, and found that the model resulted in higher predictive ability than a single-trait model considering the two pure breeds and their crosses as one population. The benefits of BOA models are therefore unclear, and it is still unknown if the results from terminal crossbreeding are transferable to rotational crossbreeding in dairy cows.

Models that account for BOA in crossbred animals rely on the accurate detection of BOA in the genotypes of crossbred animals. For F1 crosses, the genotype of the crossbred animal phased to the maternal and paternal haplotypes includes this information since the parents are purebred [11, 12]. In other cases, Vandenplas et al. [13] presented a method that can accurately detect BOA in two-way, three-way or four-way crossbred animals without requiring pedigree information to be available. However, methods for detecting BOA in rotational crossbreeding are still lacking.

Estimating local ancestry is relevant in other fields than for the calculation of GEBV of crossbreds, for example for investigating population structure [14]. Methods that assign parts of the genome to ancestor populations have been developed for these purposes [14–16]. Gajjar et al. [17] applied ChromoPainter [14] for assigning alleles to breed of origin in crosses of Holstein Friesian and native breeds in India. However, these methods and the associated software are not designed for the common crossbreeding scenarios, do not make use of pedigree information, which is usually available for dairy cattle, and are computationally slow for the typical size of datasets in dairy cattle. Thus, it is interesting to investigate whether simpler methods could assign alleles accurately in rotational crossbreeding scenarios, where pedigree information and genotypes of recent ancestors are available.

Therefore, the aims of this study were two-fold. The first aim was to develop a method for BOA assignment in crossbred dairy cows, which accounted for the available pedigree information. The second aim was to investigate if combining marker effects estimated from purebred genomic evaluations, based on estimated BOA, would result in more accurate GEBV than assuming constant breed proportions throughout the genome. The accuracy of BOA detection and reliability of GEBV were assessed on simulated data that mimicked rotational crossbreeding in dairy cattle.

## Methods

Here, first we present a method for assigning BOA for crossbred animals, where one parent is known to be purebred and the other parent can be crossbred. Second, we present methods for the calculation of GEBV of crossbred cows, where the estimated marker effects from purebred genomic evaluations are combined based on (1) the detected BOA or (2) the breed proportions estimated from the genotypes, but in both cases the records on crossbreds are not considered. Finally, we describe an application of these methods on simulated data that mimic the first four generations in a rotational crossbreeding dairy cattle program.

### Detection of breed of origin of alleles

Marker alleles of the crossbred animals were assigned to breed of origin with a new method that we name AllOr (**All**ele **Or**igin). It was designed to detect BOA in genotypes of crossbred animals from medium-density single nucleotide polymorphism (SNP) chips, where the sire is known and of a purebred known breed, as in typical rotational crossbreeding. The dam can be a purebred of a known breed or a crossbred of a number of known breeds. The genotypes of representative samples of all contributing pure breeds are required. In order to make use of the known relationships, pedigree information that connects the crossbred cows to genotyped purebred ancestors needs to be included. The method also requires breed information on each of the crossbred animals, i.e. what is the breed of the sire and which breeds are contributing to the dam, such that the program can limit the breed assignments to the possible breeds. Furthermore, the input genotypes of both the purebred and crossbred animals should be phased to two haplotypes for each animal, and these need to be complete, i.e. genotypes need to be imputed to fill in the missing ones. Phasing can be performed alongside imputation of genotypes by existing software, such as Beagle [18] or Fimpute [19]. Imputation and phasing of the genotypes of crossbred animals should be performed by including the genotypes of purebred animals in the same run.

We consider the assignment of alleles on crosses of ${N}_{b}$ breeds, numbered 1, 2,…, ${N}_{b}$, where $b$ is breed. For each crossbred animal, we have the information on which breed is the paternal breed, $bp$, and which breeds contribute to the dam, denoted ${bm}_{c}$, where $c$ can take from one to ${N}_{b}$ values, depending on how many breeds are contributing to the dam. For example, for a daughter of a sire from breed 2 and a dam which is a cross of breed 1 and 3, we have $bp=2$, ${bm}_{1}=1$ and ${bm}_{2}=3$. The assignment process consists of three steps. The first step compares the haplotypes of the crossbred animals to the haplotypes of the purebred reference animals, and the following two steps fill in unassigned loci based on the assignment of neighbouring loci and information about breed composition, respectively.

#### Assigning alleles to breed of origin

##### Step (1)

The comparison of haplotypes is performed in multiple rounds for each chromosome, each round considering a predefined window length (*WL*) of markers. The windows overlap, with a predefined number of shifted *(NS*) markers between rounds, giving an overlap of *WL*-*NS* markers. For the last window of a chromosome, the last window length (*LWL*) is *LWL* > *WL*-*NS* and *LWL* ≤ *WL*. In order to have markers at the end of chromosome included in more than one window, ${n}_{ew}$ additional rounds are added at the beginning and end of the chromosomes, with ${n}_{ew}$ being the highest integer fulfilling ${n}_{ew}$ ≤ WL*0.2/NS. The additional rounds at the beginning consider the *WL-NS*, WL-NS*2, …,WL-NS*${n}_{ew}$ first markers of the chromosome, and the additional rounds at the end of the chromosome consider the *LWL-NS*, LWL-NS*2, …, LWL-NS*${n}_{ew}$ last markers.

The most appropriate values for *WL* and *NS* in the assignment process may depend on the type of data and the required precision of breed of origin assignment. Because the windows are always shifted by *NS* markers at a time, sets of *NS* consecutive markers always fall within the same set of windows, and will thus all be assigned in the same manner in step (1). This means that a recombination which results in a shift from one breed to another in the haplotype cannot be detected at a finer scale than *NS*, and the accuracy of assignment is therefore expected to increase with smaller *NS*. However, the number of rounds and therefore running time are proportional to 1/*NS*. The optimal *WL* may depend on marker density, linkage disequilibrium and number of generations of crossbreeding.

Within each window, the two haplotypes are assumed to originate as a whole from pure breeds and are matched with haplotypes from purebred animals. Haplotypes are considered as matching if 99% or more of the marker alleles are matched, meaning that, for example for *WL* = 100, one allele is allowed to mismatch in order to consider that the haplotypes are matched.

Because all the sires are purebred, one of the two haplotypes originates from $bp$. The assignment task for the two haplotypes within each of the windows is thus two-fold, first, to determine which haplotype is the paternal haplotype and which is the maternal haplotype, and second, to determine from which breed the maternal haplotype originates. Thus, the number of possible breed assignment pairs is twice the number of possible maternal breeds. Breed assignments pairs are denoted $l=(j,{bm}_{c})$, where $j$ denotes haplotype 1 or 2 that indicates which of the two is the maternal haplotype, and ${bm}_{c}$ denotes a possible maternal breed. For each possible assignment pair, $l$, we calculate a value, ${p}_{l}$, as a measure of the probability that haplotype $j$ is the paternal allele from breed ${b}_{p}$ and haplotype $k\ne j$ is the maternal allele from breed ${bm}_{c}$. The ${p}_{l}$ value depends on whether there are matching haplotypes in the pure breeds according to $l$*.* Given that there are matching haplotypes, ${p}_{l}$ can depend on two factors: (1) on whether the matching haplotypes are present in recent purebred ancestors of the crossbred animal under consideration; and (2) on the frequency of the haplotype in the purebred populations. Therefore, ${p}_{l}$ is calculated as ${p}_{l}={pp}_{bp,j}+{pm}_{bm,k}$, where ${pp}_{bp,j}$ (${pm}_{bm,k}$) is either the relationship coefficient of the closest ancestor on the paternal (maternal) side of breed $bp$ (${bm}_{c}$) carrying the matching haplotype, traced for four generations; or, if no ancestor carries the haplotype, the proportion of matching haplotypes in the reference haplotype pool for breed $bp$ (${bm}_{c}$) restricted to ≤ 1/16, which thus does not exceed values from matching haplotypes in the pedigree. If no matches are found within breed $bp$ (${bm}_{c}$), ${pp}_{bp,j}$ (${pm}_{bm,k}$) is assigned the value zero.

Because the windows overlap, each locus is present in multiple windows. In order to obtain one assignment for each allele at each locus, we calculate the average ${p}_{l}$ value across rounds with windows that include the locus and we assign the alleles according to assignment pair $l=(j,{bm}_{c})$, i.e. allele $j$ to the paternal breed $bp$ and allele $k\ne j$ to the maternal breed ${bm}_{c}$, with the highest mean ${p}_{l}$. If no purebred haplotype matches the haplotype of the crossbred animal, i.e., all ${p}_{l}$ values are equal to zero, the alleles at the locus cannot be assigned to the breed of origin in step (1). If some of the highest ${p}_{l}$ values are the same for more than one breed assignment, $l$, then at the locus there are two possible scenarios: (1) if the same allele is considered as the paternal allele in all these assignment pairs, the paternal allele is assigned to ${b}_{p}$, but the other allele is not assigned in step (1); and (2) if the same allele is not considered as the paternal allele in all these assignment pairs, neither of the alleles at the locus are assigned in step (1).

##### Step (2)

For alleles that could not be assigned in step (1), step (2) assigns them based on neighboring assigned loci. For locus ${z}_{u}$ in haplotype $k$ with an unassigned allele, we find locus ${z}_{t}$ on the left side of ${z}_{u}$, i.e. the locus closest to ${z}_{u}$ with an assigned allele in haplotype $k$_*,*_, and locus ${z}_{v}$ on the right side of ${z}_{u}$, i.e. the locus closest to ${z}_{u}$ with an assigned allele in haplotype $k$. If ${z}_{v}-{z}_{t}$ is less than 2*WL and the alleles at ${z}_{t}$ and ${z}_{v}$ are assigned to the same breed, the alleles at loci ${z}_{u}$ are assigned accordingly. For unassigned loci ${z}_{u}$ where no ${z}_{t}$ value could be found because there are no loci with an assigned allele at the beginning of the chromosome, the alleles are assigned in the same way as for the allele at ${z}_{v}$, if ${z}_{v}$ is less than WL*0.8 markers, i.e. if it has the same length as the shortest window considered in step (1). In the same manner, for loci ${z}_{u}$ where no ${z}_{v}$ value can be found because there are no loci with an assigned allele at the end of the chromosome, the allele at ${z}_{u}$ is assigned in the same way as the alleles at loci ${z}_{t}$, if ${z}_{t}$ is less than or equal to WL*0.8 markers from the end of the chromosome.

##### Step (3)

 For homozygous loci, which allele is the maternal allele and which one is the paternal allele are not relevant. Thus, one of the alleles at homozygous loci that are still unassigned after steps (1) and (2) is assigned to $bp$. Similarly, for F1 crosses, since the dam is purebred, one of the alleles at homozygous loci is assigned to the maternal breed, ${bm}_{c}$.

##### Unassigned alleles

The alleles that are still unassigned at this point cannot be assigned to a definite breed of origin. Instead, the output of AllOr includes information about which breeds are possible based on information from the assignment process, if available, otherwise from the input to the program. Probabilities of BOA are given based on the following rules applied successively: (1) if the allele could not be assigned because of several non-zero ${p}_{l}$ values being equal in step (1), it is considered equally likely that it comes from the corresponding breeds; (2) if neighboring alleles, at ${z}_{t}$ and ${z}_{v}$, on the same haplotype strand were assigned to two different breeds, the corresponding breeds are considered equally likely as breed of origin of the unassigned allele; (3) if one allele at a locus was assigned to the paternal breed in step (3), the other allele is considered equally likely to originate from any of the possible maternal breeds; and (4) if both alleles at a locus are unassigned and rules (1) to (3) do not apply, the paternal breed is assumed to have a probability of 0.5 and each maternal breed is assumed to have a probability of 0.5 divided by the number of possible maternal breeds. For F1 and three-way crosses, these are the expected breed proportions, but for crossbreds from more generations of crossbreeding, BOA proportions of unassigned loci are not expected to equal the expected breed proportions because breeds with a larger contribution are expected to be represented by longer haplotypes in the genome of the crossbred, and thus, BOA assignment using the AllOr method is expected to be more effective for the alleles from these breeds. In other words, alleles from such breeds are less likely to be unassigned. For simplicity, it is assumed that BOA proportions for unassigned loci have equal probabilities of contributing maternal breeds.

#### Implementation

AllOr was implemented in a Fortran program to test the method on simulated data for which breed of origin of alleles was known. The program runs on one chromosome at a time. It was not optimized for speed, thus improvement regarding speed could be made. All the results presented here are from runs with *NS* = 5. Three values of *WL*, 100, 150 and 200, were tested on one replicate of the simulated data (as explained later), and based on the results obtained, *WL* = 100 was used for further analysis.

### Prediction models

We used two genomic prediction models that are adapted for prediction of crossbreds: (1) the breed of origin model (BOM), where marker effects estimated from the evaluation of purebreds are combined based on assigned BOA; and (2) the breed proportion model (BPM), where marker effects estimated from the evaluation of purebreds are combined using BBR, i.e. the estimated contributions of pure breeds to a crossbred genotype are used as weights.

The GEBV for animal $i$ from the BOM model is calculated as:

$${GEBV}_{BOM,i}=\sum_{b=1}^{{N}_{b}}({\mathbf{v}}_{\mathbf{b}}^{{\mathbf{\prime}}}{(\mathbf{w}}_{\mathbf{i},\mathbf{1}}\circ {\mathbf{s}}_{\mathbf{1},\mathbf{i},\mathbf{b}})+{\mathbf{v}}_{\mathbf{b}}^{{\mathbf{\prime}}}({\mathbf{w}}_{\mathbf{i},\mathbf{2}}\circ {\mathbf{s}}_{\mathbf{2},\mathbf{i},\mathbf{b}}))+{\upmu }_{b}\frac{\sum {\mathbf{s}}_{\mathbf{1},\mathbf{i},\mathbf{b}}+\sum {\mathbf{s}}_{\mathbf{2},\mathbf{i},\mathbf{b}}}{2m},$$

where ${\mathbf{w}}_{i,j}$ contains haplotype $j$ of animal $i$ coded as 0 and 1 for the alternative alleles; $\circ$ is an element-wise multiplication; ${\mathbf{v}}_{b}$ is a vector of estimated marker effects for breed $b$, ${\mathbf{s}}_{j,i,b}$ is a vector of breed $b$ origin indications for alleles in haplotype $j$ of animal $i$, with 1 for alleles assigned to breed $b$, 0 for alleles assigned to other breeds, and values between 0 and 1 for alleles that could not be assigned to a definite breed, according to the description of the AllOr method in the previous subsection; ${\upmu }_{b}$ is the estimated intercept for breed $b$; $\sum {\mathbf{s}}_{j,i,b}$ is the sum over $b$ of all the elements in ${\mathbf{s}}_{j,i,b}$, for $j$ = 1,2; $m$ is the number of markers; and ${N}_{b}$ is the number of breeds.

The BPM model is based on the methods presented by VanRaden et al. [9]. BBR was calculated from the genotypes as in VanRaden et al. [9], but with some differences as described here. First, genomic breed composition (GBC) was estimated from all marker genotypes as in VanRaden et al. [20], but with a linear Gaussian model rather than assuming a heavy-tailed distribution of marker effects. Marker effects to predict GBC of breed $b$ = 1, 2,…, ${N}_{b}$ were estimated with the model: ${\mathbf{bc}}_{b}={\mathbf{1}}^{\mathbf{\prime}}{k}_{b}+{\mathbf{Zg}}_{b}+{\mathbf{e}}_{b}$, where ${\mathbf{bc}}_{b}$ is a vector of known breed compositions for the purebred animals, i.e. ${bc}_{b,i}$ = 1 for purebred animal $i$ from breed $b$, and ${bc}_{b,i}$ = 0 for animal $i$ from another breed; $\mathbf{Z}$ is an $n\times m$ matrix of allele content of $n$ animals for $m$ markers; ${\mathbf{g}}_{b}$ is the vector of marker regression coefficients for breed $b$ with variance $\mathbf{I}{\sigma }_{b}^{2}$, ${\sigma }_{b}^{2}$ being 99% of the variance of ${\mathbf{bc}}_{b}$*,* ${\sigma }_{bc}^{2}$; **1** is a vector of 1 s; ${k}_{b}$ is an intercept for breed $b$; and ${\mathbf{e}}_{b}$ is a vector of random residuals, with variance ${\mathbf{I}}{\sigma }_{e}^{2}$, ${\sigma }_{e}^{2}$ being 1% of ${\sigma }_{bc}^{2}$. Note that the absolute value of ${\sigma }_{bc}^{2}$ is irrelevant because it cancels out in the mixed model equations. Genomic breed compositions for breed $b$ in crossbred animals were calculated as ${GBC}_{b}={k}_{b}+{\mathbf{Zg}}_{b}$. Such estimates for individual breeds can be lower than 0 for breeds with no or very little contribution to the animal, or higher than 1 if an animal is, in fact, purebred. Thus, such estimates for individual animals were converted to BBR values between 0 and 1, as described in VanRaden et al. [9].

The GEBV for animal $i$ from the BPM model is calculated as:

$${GEBV}_{BPM,i}=\sum_{b=1}^{{N}_{b}}{BBR}_{b,i}\left({\upmu }_{b}+{\mathbf{z}}_{i}^{\mathbf{{\prime}}}{\mathbf{v}}_{b}\right),$$

where ${BBR}_{b,i}$ is the BBR value for breed $b$ in animal $i$, ${\mathbf{z}}_{i}$ is a vector of allele contents for animal $i$ and other terms are as described for BOM.

### Simulations

For assessing the BOA assignment using the AllOr method and the prediction reliability of the prediction models for crossbred cows, we simulated genotypes, breeding values, and phenotypes of three dairy cattle breeds, Holstein (HOL), Jersey (JER) and Red Dairy Cattle (RDC), and their crosses in four generations of a rotational crossbreeding system as explained below. The simulations were performed with self-written Julia [21] scripts, based on the simulation tools described in Karaman et al. [22]. Subsets of 2100 animals genotyped with the 50K SNP chip from each of the three dairy cattle populations, Danish Holstein, Danish Jersey and Swedish Red, were used as base populations to simulate three pure breeds, HOL, JER and RDC, respectively, and their crosses (CRS) for four non-overlapping generations (G1 to G4). For computational reasons, only the 13,324 SNPs on the first five chromosomes were considered. Simulations started by randomly assigning 100 animals as males and 2000 as females in these base populations (G0) of each breed, and this male:female ratio of 1:20 was kept constant throughout the simulations. Among the 100 available males, 100 sires were selected at random with replacement, and each one was mated with 20 dams, 19 of which produced one offspring and one produced two offspring. No selection was applied for the dams and all 2000 females were used. The purebred sires were mated with dams either from their own breed to produce purebred offspring or from another breed to produce crossbred offspring, when simulating the next generation of purebred and crossbred populations (as explained below). The selection of sires was done separately for purebreds and crossbreds, and therefore, sires used for pure breeds and those used for CRS may not fully overlap in each generation. The simulation of G1 for CRS (CRS_G1) was achieved by mating sires from the JER base population (JER_G0) with dams from the HOL base population (HOL_G0). Crosses of sires of RDC_G1 and dams of CRS_G1 formed the second generation of CRS (CRS_G2). Similarly, sires of HOL_G2 and dams of CRS_G2 were mated to produce the third generation of CRS (CRS_G3). The CRS_G4 were the offspring of CRS_G3 dams and JER_G3 sires. An overview of the simulated crossbreeding program is in Table 1.

**Table 1:** Overview of the simulated mating program for the rotational crossbreeding

Generations	Sires	Dams
G1	JER_G0	HOL_G0
G2	RDC_G1	CRS_G1
G3	HOL_G2	CRS_G2
G4	JER_G3	CRS_G3

Parents of the crossbred animals across the four simulated generation

Among the available 13,324 SNPs, 250 were selected at random from those that had a minor allele frequency (MAF) within a 0.01 to 0.30 range, with MAF being calculated from the averages of allele frequencies computed separately for each breed at G0. These 250 SNPs were assigned to be QTL and were then excluded from the final set of SNPs before the analyses. Allele substitution effects of the QTL were assumed to be identical across the breeds, and were sampled from the standard normal distribution. These substitution effects were then multiplied by the number of reference alleles (0, 1, or 2) at each locus to compute true breeding values (BV) for purebred and crossbred animals. Since allele frequencies of QTL differed among breeds, the genetic variances (variances of true BV) also differed among breeds. The substitution effects of QTL were scaled such that the mean genetic variance across the three pure breeds was 100 in G0. A residual simulated from a normal distribution $\sim (N\left(0,{\sigma }_{e}^{2}\right)$) was added to the BV of an animal to form its phenotype, and the size of ${\sigma }_{e}^{2}$ was determined by the heritability of the trait, which was 0.4 at G0. The heritabilities of traits varied around 0.4 for the individual breeds at G0, due to the use of a single value of ${\sigma }_{e}^{2}$ for all breeds. The simulation was similar to that described in more detail in Karaman et al. [22].

Within each pure breed, genotypes from a set of animals were selected as reference genotypes for estimating marker effects for BBR estimation and for comparison of haplotypes to detect BOA. The reference set consisted of purebred bulls from generations G0, G1, G2 and G3 and 10% of the cows from the same generations, resulting in 1200 animals from each breed. Although the true phase of the simulated genotypes was known, in order to make the testing of the AllOr method and the prediction models more realistic and to include possible phasing errors, the genotypes were phased using Fimpute [18] including the genotypes of purebreds and crossbreds.

Marker effects for GEBV were estimated for each pure breed separately using phenotypes and genotypes of the purebred cows from generations G0, G1, G2 and G3, with 8000 individuals from each breed. A SNP-BLUP model for the estimation of marker effects was used:

$${\mathbf{y}}_{b}={\mathbf{1}}^{{\mathbf{\prime}}}{\upmu }_{b}+{\mathbf{Z}}_{b}{\mathbf{g}}_{b}+\mathbf{e},$$

where ${\mathbf{y}}_{b}$ is a vector of phenotypes of cows from breed $b$; ${\mathbf{1}}$ is a vector of 1 s; ${\mathbf{Z}}_{b}$ is a matrix of allele content of the purebreds of breed $b$; ${\mathbf{g}}_{b}$ is a vector of random effects of markers for breed $b$ following a normal distribution $\sim N\left(\mathbf{0},{\mathbf{I}\sigma }_{g}^{2}\right)$, where ${\sigma }_{g}^{2}$ is the true genetic variance divided by $\sum 2{p}_{j}{(1-p}_{j})$ with ${p}_{j}$ being the MAF of locus $j$; and $\mathbf{e}$ is a vector of random residuals following a normal distribution $\sim N\left(\mathbf{0},\mathbf{I}{\sigma }_{e}^{2}\right)$. The marker effects were estimated using the dmu4 module of the DMU package [23], given true variances. Breed of origin of alleles, BBR and GEBV calculated with both BOM and BPM were calculated for the 8400 animals, 2100 in each generation, in CRS_G1, CRS_G2, CRS_G3 and CRS_G4, using the marker effects and intercepts that were estimated in the purebreds, and estimated BOA and BBR.

All the steps in the simulation were repeated 10 times. Although the simulations used the same set of base populations, and therefore, the number of SNPs that could be selected as QTL was fixed, the selection of SNPs as QTL was independent across replicates resulting in limited overlap between QTL in different replicates. Similarly, the animals at G0 were randomly assigned as males and females for each replicate of simulated data.

## Results

### Detection of breed of origin

The detection of BOA for chromosome 1 that harbored 3270 markers took around ten hours using the AllOr program for the 8400 simulated crossbred animals. Investigation of different values of *WL* on one replicate over the five chromosomes showed that 99.6% of the alleles were correctly assigned using *WL* = 100, but 99.2% and 98.8% using *WL* = 150 and *WL* = 200, respectively. The number of incorrectly assigned alleles was also larger with longer window lengths, i.e. 0.3% and 0.5% using *WL* = 150 and 200 respectively, compared to 0.2% using *WL* = 100.

**Fig. 1:**
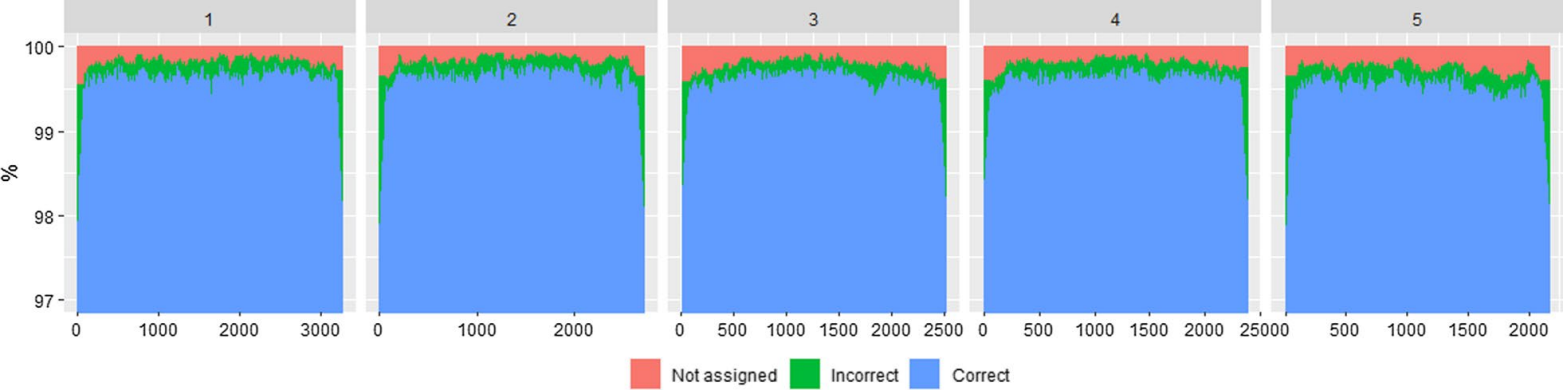
Average breed of origin assignment across markers. Average proportion of alleles correctly, incorrectly and not assigned to breed of origin for the first five chromosomes in the first four generations of simulated rotational crosses of Holstein, Jersey and Red Dairy Cattle using the AllOr method

Figure 1 shows, for one replicate, the assignment results across the five chromosomes considered here; the accuracy of BOA detection was poorest close to the ends of chromosomes, i.e. as low as 97.7% of the alleles were assigned to the correct breed of origin and up to 2.0% of the alleles were incorrectly assigned (in both cases, it concerned the first marker of chromosome 5). For the animal (from CRS_G2) with the lowest percentage of correct BOA, 89.0% of the alleles were correctly assigned, but 10.8% were not assigned to a breed of origin. In this replicate, only this animal had less than 90% of its alleles correctly assigned, and in total only 0.1% of the simulated crossbred animals had less than 95% of their alleles correctly assigned.

Across the ten replicates and four types of crosses, BOA could be correctly assigned for 99.6% of the alleles (Table 2), 0.2% were incorrectly assigned, and 0.2% could not be assigned to a specific breed. One hundred percent of the assignments to breed of origin were correct for the alleles of the animals in CRS_G1, which are F1 crosses, whereas for the more complicated crosses, 0.2 to 0.3% of the alleles were incorrectly assigned, and 0.2 to 0.3% were not assigned to a breed of origin.

**Table 2:** Breed of origin assignment

Group	N	Correct	Incorrect	Not assigned
CRS_G1	2100	100.0%	0.0%	0.0%
CRS_G2	2100	99.5%	0.2%	0.3%
CRS_G3	2100	99.4%	0.3%	0.3%
CRS_G4	2100	99.5%	0.3%	0.2%
All	8400	99.6%	0.2%	0.2%

Percentage of alleles on the first five chromosomes in the first four generations (CRS_G1-CRS_G4) of simulated rotational crossbreeding of Holstein, Jersey and Nordic Red Cattle cows that were correctly, incorrectly, or not assigned to breed of origin using the AllOr method

### Breed base representation

The estimated values of GBC summed to 100% and the only correction to calculate BBR was for CRS_G1 for which the RDC contribution was estimated to be negative in some cases. Table 3 shows both the true breed proportions and estimated BBR. For all types of crosses, RDC proportions are slightly overestimated and HOL proportions are slightly underestimated on average. Average absolute errors were lowest for the JER proportion in CRS_G1, 0.5%, and highest for the RDC proportion in CRS_G2, 1.5%. The JER proportion was the most precisely estimated proportion in all groups.

**Table 3:** Breed base representation

	CRS_G1	CRS_G2	CRS_G3	CRS_G4	All
True GBC					
Holstein	50.0%	25.0%	62.5%	31.3%	42.2%
Jersey	50.0%	25.1%	12.5%	56.2%	35.9%
RDC	0.0%	50.0%	25.0%	12.4%	21.9%
BBR					
Holstein	49.6%	24.8%	62.1%	31.1%	41.9%
Jersey	50.1%	25.1%	12.6%	56.3%	36.0%
RDC	0.6%	50.1%	25.3%	12.6%	22.1%
Absolute difference					
Holstein	0.9%	1.2%	1.4%	1.3%	1.2%
Jersey	0.5%	0.9%	0.8%	0.7%	0.7%
RDC	0.6%	1.1%	1.5%	1.3%	1.1%

Mean true genomic breed proportion (GBC) based on the true origin of all markers across five chromosomes, mean estimated base breed representation (BBR), and mean absolute difference of true and estimated GBC for the first four generations of rotational crossbreeding

### Prediction

Reliability of GEBV was highest for CRS_G1 (F1 crosses), both with BOM and BPM, and lowest for CRS_G4 (Table 4), which was the group of crossbreds that had the largest number generations of crossbreeding. The BOM model resulted in more reliable predictions than BPM for all crosses. The difference in reliability was largest for CRS_G1, i.e. 0.097, but smallest for CRS_G3, i.e. 0.054. The reliability of the average GEBV of purebred parents for CRS_G1 was 0.396, which is lower than the reliabilities obtained with either BOM or BPM.

**Table 4:** Reliability of genomic estimated breeding values

Group	CRS_G1	CRS_G2	CRS_G3	CRS_G4	All
BOM	0.73 (0.01)	0.62 (0.02)	0.62 (0.02)	0.59 (0.02)	0.65 (0.02)
BPM	0.64 (0.01)	0.56 (0.02)	0.57 (0.02)	0.52 (0.02)	0.58 (0.02)
PA	0.40 (0.02)				

Reliability of genomic estimated breeding values estimated as the squared correlation between true breeding value and estimated breeding value from the breed of origin model (BOM), breed proportion model (BPM), and average of purebred parents (PA). Standard deviations of reliability estimates across the 10 replicates are presented in brackets

## Discussion

We present a novel method that is simple and accurate for detecting BOA in crossbred dairy cows in a rotational crossbreeding scheme when pedigree information is available. We also show with simulated data that taking BOA into account in a genomic prediction model results in more accurate predictions for crossbred cows than predictions obtained by accounting for estimated genomic breed proportions in a setup where training was on purebred animals only.

### Detection of breed of origin

The proportions of alleles correctly assigned to breed of origin (Table 2) were higher than those reported by Vandenplas et al. [13] for F1 and three-way crosses. In the rotational crossing system that was investigated here, we always had purebred sires. Therefore, we always had one haplotype, i.e., the paternal haplotype, originating as a whole from one breed, which greatly simplifies the detection of BOA. However, the maternal haplotype could be a mosaic of up to three breeds, although on average half of this haplotype originated from the breed of the maternal grandsire. The proportion of correctly assigned BOA was similar in CRS_G2, CRS_G3 and CR_G4, which indicates that the method was not sensitive at least up to four generations of rotational crossing on the maternal side. The method of Vandenplas et al. [13] does not consider pedigree information, which is a very important source of information in our method. It is especially useful when genotypes on the sires are included in the reference genotypes, as was the case in our study.

The AllOr Fortran program took up to ten hours to run on the largest chromosome (that harbored ~ 3270 SNPs) for the 8400 simulated crossbred animals. However, there are many possibilities to increase speed, for example by saving the information about matching haplotypes across overlapping windows, constructing a haplotype library of unique purebred haplotypes in order to avoid comparing the crossbred haplotype with the same common haplotype many times, and parallel computing. We did an additional trial with ChromoPainter [14] and the results showed that the running time of AllOr was still much shorter than that of ChromoPainter for which the processing of chromosome 1 with the same number of genotypes of crossbred animals and same number of purebred reference genotypes took about two weeks. However, the accuracy of breed assignment was higher for ChromoPainter for which 99.9% of the alleles were correctly assigned to breed of origin. Thus, for a relatively smaller number of BOA detection tasks and when a high accuracy is required, ChromoPainter seems to be a slightly better choice than AllOr.

The overlapping window approach adopted in AllOr is similar to that applied in the LAMP software [15]. However, combining assignments across rounds is based on an average ${p}_{l}$ value in AllOr, whereas in LAMP the combination is based on the most common assignment across rounds, and therefore does not take differences in confidence of the assignment of the windows in each round into account. In addition, the within-window assignment differs greatly between these methods. In LAMP, the window size is decided based on the number of generations of admixture, number of populations, and recombination rate [15], and it is claimed that its length should be sufficiently short so that there is nearly no recombination sites within each window. The same is true for *WL* in AllOr, in which the windows should be sufficiently short to ensure that recombination between breeds is rare within each window. With more generations of crossbreeding in the ancestry of the crossbred animals, *WL* should be shorter, but shorter haplotypes are more likely to exist in more than one breed [24], which can lead to errors in BOA assignment. However, the three *WL*, i.e. 200, 150 and 100 markers, tested in this study, did not show more errors with shorter windows. The *WL* values tested here were within the range, or smaller, than those tested by Vandenplas et al. [13] (150–350) as core lengths for their program and phasing with AlphaPhase [25] for F1 and three-way crosses in pigs. Contrary to their approach, the running time of AllOr is shorter with shorter *WL* because the number of rounds in the first step of AllOr only increases marginally with longer *WL*, but the number of comparison within rounds increases with longer *WL*. Figure 1 clearly shows that the efficiency of AllOr was lowest for loci close to the extremes of the chromosomes. This was expected because BOA of haplotype segments, which result from recombination that is located less than *WL* markers from either end of the chromosome, cannot be detected with AllOr, although this is partially compensated by including extra rounds with a shorter *WL* at the ends of the chromosomes. The performance of any haplotype-based assignment method will be poorest at the chromosome extremes because the number of possible matching haplotypes is smaller when only one direction can be considered.

AllOr, like Chromopainter [14] and the BOA program of Vandenplas et al. [13], relies on the accurate phasing of the genotypes obtained with an external software. Vandenplas et al. [13] took possible inaccuracies in phasing into account by using the output of multiple rounds of AlphaPhase [25]. In principle, phasing methods using pedigree information [19] or long-range phasing [25, 26] do identify haplotype segments, which the animals have inherited from ancestors. Breed of origin assignment can be viewed as an extension of this task, that is, if similarities of haplotypes between the crossbred and purebred animals are used for phasing the genotype of the crossbreds, information on the breed of the purebred animal could be stored. This information indicates breed of origin of the haplotype in the crossbreds. Phasing and BOA detection would thus be performed jointly in one step, instead of identifying the matching haplotypes again in a separate BOA detection procedure as we do here. Extending a phasing and imputation software to include BOA assignment of crossbred animals could be an interesting topic for future research.

In spite of our effort to simulate a realistic situation, applying AllOr on real genotypes creates additional challenges. For example, genotype errors were not included in the simulations. Allowing some mismatches in the comparison of haplotypes, here a 1% mismatch was set, should allow assignment in many cases in spite of sporadic genotyping errors. In addition, potential errors in pedigree information and the presence of only a few or no genotyped ancestors could further complicate BOA assignment for individual animals. The BOA program of Vandenplas et al. [13] showed a higher proportion of alleles in real genotypes that could not be assigned than in most of the simulated scenarios that they studied. Application of their program on three-way pig data assigned 95.2% of the alleles to breed of origin [10], but on chicken data 91.8% of the alleles could be assigned to breed of origin for the three-way crosses and 96.9% for the F1 crosses [27]. Our preliminary results of the application of AllOr on real genotypes of crossbred dairy cows are promising with more than 99% of the alleles assigned to breed of origin (personal communication).

### Genomic predictions

Comparison of GEBV with parent averages was only possible for CRS_G1 because, for the other groups, both parents were not purebred. Higher reliability of genomic predictions than of parent averages (Table 4) was expected and in agreement with multiple previous studies, e.g. VanRaden et al. [28], Su et al. [29] and Bengtsson et al. [30]. For crossbred dairy cows, VanRaden et al. [9] found that genomic prediction of breeding values of crossbred dairy cows with the breed proportion model was more accurate than parent averages for seven of the eight traits tested.

The reliability of GEBV obtained with both BOM and BPM was highest for CRS_G1, i.e. the F1 crosses (Table 4) and lowest for CRS_G4, i.e. the crosses that had the largest number of generations of crossbred ancestors. The reliability of GEBV using these methods is expected to drop for crossbreds with more generations for several reasons. First, in the F1 crosses, the maternal and paternal haplotypes originate as a whole from their purebred parents, and thus the linkage between markers and QTL in the pure breeds is fully consistent. In CRS_G2, this linkage starts to break in the maternal haplotype. In CRS_G3, an expected quarter of the genome originates from a crossbred grand-dam and has more breakage of linkage compared to CRS_G2, resulting in a further expected reduction of reliability. However, the degree of this reduction is smaller than between CRS_G1 and CRS_G2, because the effect is on an expected quarter of the genome compared to an expected half between CRS_G1 and CRS_G2. Similarly, one eighth more of the genome is affected between CRS_G3 and CRS_G4. This could explain partly why the drop in reliability is much larger between CRS_G1 and CRS_G2 than between CRS_G2 and CRS_G3, and between CRS_G3 and CRS_G4. An additional factor is the segregation variances in three-way crosses, and in crossbreds which have more generations of crossbreeding [31]. For CRS_G2-CRS_G4, the differences in allele frequency between breeds contribute to the genetic variation, and in our study, this variation was not captured in the estimated marker effects because training was only within pure breeds. The inability to fully predict the variability in breeding values related to segregation variances could have affected the reliability in CRS_G2-CRS_G4, which could also contribute to the drop in reliability observed from CRS_G1 to CRS_G2. Another factor, which could have affected the reliabilities of predictions for different groups, was the difference in linkage disequilibrium levels between the breeds. Because the starting point of the simulations was real genotypes from the three breeds, differences in linkage disequilibrium between the breeds should be present in the simulations. The population structure of RDC is more complicated than that of JER and HOL [32], and it could have reduced the reliability of predictions of groups in which a high proportion of the genome originated from RDC. In order to investigate if this was the case, we checked the reliability of prediction for purebred animals from HOL_G4, JER_G4 and RDC_G4, using estimated marker effects from the relevant breed. The result showed that the reliability was lowest (0.66) for RDC_G4, compared with 0.71 for HOL_G4 and 0.69 for JER_G4, which indicates that the estimated marker effects for RDC did not result in predictions as reliable as for the other breeds. In CRS_G2, RDC is the paternal breed with a 50% contribution, and thus the lower reliability of prediction from RDC marker effects could have contributed to the relatively low reliability for CRS_G2 compared to CRS_G1 and CRS_G3, where the RDC proportion was 0 and 25% respectively, in addition to the factors mentioned previously.

Our results indicate that BOM is superior to BPM for the prediction of genetic merit of crossbred cows for the first generations of rotational crossbreeding between HOL, JER and RDC. In CRS_G1 and CRS_G2, all heterozygous loci had alleles from two different breeds. For CRS_G3 and CRS_G4 75% and 87.5%, respectively, of the loci were expected to have alleles from different breeds. Among those loci, BOA was relevant for the heterozygous loci. Assuming correct BOA and correct BBR for the F1 crosses, the SNP estimates obtained with BOM and BPM are the same for homozygous loci but differ by half the difference in allele effects from the two breeds for heterozygous loci. To what extent the marker effects differ between breeds is therefore an important factor to determine if BOA should be accounted for as shown by Ibánẽz-Escriche et al. [4]. Although we simulated the same QTL effects across breeds, the estimated marker effects differed considerably across breeds because of differences in allele frequency and linkage between QTL and markers. The correlation between GEBV for crossbred animals, which was calculated with the estimated marker effects for HOL and JER respectively, was 0.07 on average across the replicates, 0.14 between HOL and RDC, and 0.07 between JER and RDC. These differences reflect the relatedness between the breeds since the base generations of the purebreds (G0) in the simulations were real genotypes from the breeds [22]. In CRS_G2, the marker alleles at approximately half of the loci were from HOL and RDC, and at the other half from JER and RDC. The smaller difference in estimated marker effects between HOL and RDC compared to that between HOL and JER explains the smaller difference between BOM and BPM for CRS_G2 compared to CRS_G1. Over the four generations investigated, it was apparent that the benefit of BOM over BPM was largest for CRS_G1 and CRS_G4, in which the sires were purebred JER, which underlines the importance of how breeds are related in such a comparison.

In addition to accounting for the origin of the two alleles at heterozygous loci, BOM also accounts for variation in breed proportions across the genome, contrary to BPM. The breed proportions were constant throughout the genomes for CRS_G1 but varied for other crosses, depending on local ancestry, which should benefit BOM compared to BPM. However, the difference in the reliability of the models was largest for CRS_G1, which indicates that other factors, such as the similarity of the breeds in question, were more important in this case.

Errors in the detected BOA will impair the reliability of prediction with BOA models [11, 12], especially if the breeds are distantly related [4]. However, breed of origin detection is more accurate in less related breeds [13], which results in a lower risk of errors. In our study, the low correlation between marker effects makes correct assignment important but BOA assignment was very accurate (Table 2). We investigated the effects of incorrect or missing BOA assignments in an additional analysis, in which we used the BOM model with true BOA rather than estimated BOA. The results showed that the reliability was not higher using true BOA, which indicates that inaccuracies in BOA detection did not affect reliability when they were as rare as in our study. Alleles that are not assigned to a definite breed have less effect on the prediction than incorrectly assigned alleles. In their analysis, Sevillano et al. [10] set loci with alleles that were not assigned to a breed as missing. However, we considered that including all markers was a better approach, and we included the effects proportionally by breeds if BOA could not be detected. As long as the correlations between estimated marker effects from purebreds are positive, including markers regardless of whether BOA is incorrect or undetermined should be better than not including them. Thus, with the BOM model, including all the markers should increase reliability compared to excluding the markers with undetermined BOA.

When training is done separately in different purebred populations, we cannot detect genetic variation that exists only between breeds and not within breeds. This variation is therefore not included in the estimated marker effects and we cannot predict this part with the BOM or BPM models presented here. In particular, when an allele at a QTL is fixed in one breed, its effect is not estimated in this breed, and because it is common to all animals of the breed, it is included in the intercept. Thus, BOM cannot determine if a crossbred animal has markers connected to the allele and the effect of the allele will be included according to the proportion of the breed via the intercept, ${\upmu }_{b}$. Training on crossbreds would be necessary to account for alleles fixed in alternating states and for different fixed loci in two breeds. Future developments of prediction models should investigate how this is best done in rotational crossbreeding. In situations where only the breeding value of the purebred is of interest, as in terminal crossing systems [4, 10, 11], this is not relevant because the selection is within pure breeds.

It is well established in terminal crossbreeding systems that the genetic correlation between crossbred performance and purebred performance is lower than 1 [33–35]. The difference between purebred and crossbred performance should be accounted for in prediction models where both purebred and crossbred records are included [10, 11, 33]. In this study, we included only the phenotypes of purebred, and thus the predictions were for purebred performance. In the simulation, QTL effects were the same in crossbreds and purebreds, and thus it did not take the possible effect of different genetic backgrounds on the effects of QTL in purebred and crossbred into account. In rotational crossbreeding, the crossbred group is not uniform, and therefore a large number of traits would have to be defined if each breed combination was defined as a separate trait. Including dominance effect in the model to account for heterosis may be a more attractive approach, and for example Esfandyari et al. [36] proposed a model where dominance is included when training purebreds for the prediction of crossbreds.

### Breed averages

The benefits of using the method of VanRaden et al. [9] and the BOM and BPM models presented here are that the estimated marker effects that are already available from genomic evaluations of purebred animals and the large reference populations that have been established for many purebred populations can be used. In this study, the intercepts for correcting for potential differences in genetic level of the breeds were available from the estimation of marker effects. For genomic evaluations with more complicated models, and for example for which the input data are deregressed proofs or yield deviations from pedigree analyses rather than direct phenotypes, informative intercepts might not be available. The genetic evaluation that was the basis for the estimated marker effects in VanRaden et al. [9] was from a multi-breed genetic evaluation, which made the estimated marker effects comparable between breeds. In the case of separate genomic evaluations for each breed, a possible solution is to approximate breed differences by taking the phenotypic means of a group from each of the breeds in production environments that are similar across breeds, and subtract the mean direct genomic value of the same group using the estimated marker effects from the relative breed. With an increased number of genotyped crossbred cows, an alternative approach could be to determine the intercepts from phenotypes and genotypes on crossbreds with a varying proportion of purebreds and a correction for heterosis.

## Conclusions

In the presence of reliable pedigree information and extensive genotyping of purebred sires, marker alleles of crossbred cows from rotational crossbreeding can be detected accurately using the AllOr method. Taking BOA into account can facilitate genomic prediction of crossbred cows by using marker effects estimated from the genomic evaluation of purebreds, and our results indicate an advantage of BOM over BPM. The BOM model could be an interesting option for providing GEBV for crossbred dairy cows without having to establish a large reference set of crossbreds. Thus, it could contribute to more efficient dairy farming, where the benefits of the genomic prediction of cows and crossbreeding can be used simultaneously.

## Data Availability

The datasets supporting the conclusions of this article and the AllOr program are available from the first author upon reasonable request.
